# Localized Corrosion by Chromium Nitride Precipitation in Low-Temperature Plasma-Nitrided Inconel 718

**DOI:** 10.3390/ma19010063

**Published:** 2025-12-23

**Authors:** Juan Fernando Uribe Cruz, Oriana Palma Calabokis, Vladimir Ballesteros-Ballesteros, Yamid E. Nuñez de la Rosa, Edward Andrés Gil González

**Affiliations:** 1 Faculty of Engineering and Basic Sciences, Fundación Universitaria Los Libertadores, Bogotá 111221, Colombia; jfuribec@libertadores.edu.co (J.F.U.C.); vladimir.ballesteros@libertadores.edu.co (V.B.-B.); yenunezd@libertadores.edu.co (Y.E.N.d.l.R.); eagilg@libertadores.edu.co (E.A.G.G.); 2 Soft Matter Research Center—SOFMAT, Research Center for Rheology and Non-Newtonian Fluids—CERNN, Federal University of Technology–Parana—UTFPR, Curitiba 81280-340, Paraná, Brazil

**Keywords:** chloride environments, chromium nitride CrN, corrosion resistance, critical pitting temperature, Inconel 718, plasma nitriding

## Abstract

Inconel 718 is widely used in chloride-bearing environments where localized corrosion resistance is critical. This study assesses the effect of continuous low-temperature plasma nitriding (425 °C, 2 h) on the microstructure, hardness, and localized corrosion behavior of Inconel 718. The nitriding treatment produced a surface layer with hardness values up to three times higher than those of the untreated material, associated with a nitrided layer of thickness 6.1–6.7 µm. X-ray diffraction confirmed the precipitation of CrN without the formation of nitrogen-expanded phases. Cyclic polarization tests revealed non-significant changes in the corrosion parameters, except for a two-fold increase in the corrosion rate of nitrided samples. Also, the critical pitting temperature (CPT) decreased by more than 30 °C on average in the nitrided condition, falling below 10 °C. These findings indicate that, although continuous plasma nitriding enhances surface hardening, it significantly compromises the alloy’s resistance to localized corrosion in chloride-rich environments.

## 1. Introduction

Inconel 718 is widely recognized for its excellent corrosion resistance and its ability to maintain high mechanical strength under demanding service conditions, making it indispensable in aerospace, petrochemical, and marine environments [1,2]. Despite its robustness, the alloy remains susceptible to localized corrosion, particularly pitting and crevice attack, in chloride-rich media, a limitation that continues to challenge its long-term durability [3,4].

For that reason, surface engineering has become an important complement to the alloy’s intrinsic properties. Techniques such as boriding, carburizing, and nitriding have been explored over the years [5,6,7]. Among them, plasma nitriding has gained prominence for nickel-based alloys, including Inconel 718, due to its ability to introduce nitrogen into the surface, forming hardened layers, and inducing beneficial microstructural modifications [8]. Compared with gas or salt-bath nitriding, plasma nitriding offers improved control over layer thickness, reduced thermal distortion, shorter treatment times, and lower environmental impact [9].

However, the success of plasma nitriding depends strongly on processing temperature, time, and nitrogen potential. At low temperatures, typically around 400 °C, nitrogen incorporation can produce an expanded austenite layer (γN or S phase), which generally enhances both hardness and corrosion resistance [10,11]. As temperature and exposure time increase, chromium diffusion becomes more significant, and chromium nitride (CrN) precipitation is promoted [12,13,14]. The associated chromium depletion in the matrix destabilizes the passive film in chloride environments and introduces a compromise between mechanical strengthening and corrosion performance [13].

Recent investigations illustrate how subtle changes in process parameters shift the balance between γN formation and CrN precipitation. Kovaci et al. reported that treatments up to 600 °C increase hardness but promote brittle CrN rich surface layers with inferior corrosion resistance [15]. Maniee et al. showed that plasma nitriding near 450 °C can still favor γN formation and improve both hardness and corrosion resistance when parameters are carefully controlled, whereas higher temperatures or more severe conditions promote CrN formation and passive film breakdown [12]. Mondragón Rodríguez et al. observed that, even at 450 °C, longer nitriding times encourage CrN precipitation [16]. Similarly, Xue et al. noted improved wear and erosion corrosion resistance at 500 °C, but cautioned about long term chromium depletion [17]. In contrast, Nuñez de la Rosa et al. showed that treatments near 400 °C enhance resistance to pitting and crevice corrosion, whereas conditions at 450 °C produced layers containing γ + CrN with compromised localized corrosion resistance [4].

Studies published between 2015 and 2025 reinforce this temperature sensitivity. Low-temperature plasma nitriding of Inconel 718 consistently produces thin γN layers with substantial hardness increments when temperatures remain close to 400 °C [11,13]. At intermediate regimes, around 450 °C, γN may coexist with finely dispersed CrN, which can offer favorable mechanical behavior but leads to increasingly complex and sometimes inferior corrosion responses [18]. Phase transformation studies confirm that transformation of γ into γN, and γN + CrN occur progressively as nitrogen uptake, diffusion kinetics, and temperature interact [19]. A recent review concluded that Inconel 718 achieves the best compromise between hardness, roughness, and corrosion resistance when γN dominated layers are obtained by carefully controlled low temperature nitriding, keeping CrN precipitation limited [14].

Collectively, the literature shows that plasma nitriding of Inconel 718 can significantly enhance mechanical properties, but its effect on corrosion resistance remains highly sensitive to process control. Given these open questions, this study focuses specifically on continuous low temperature plasma nitriding at 425 °C for 2 h, a condition located in a transition zone where the competition between γN and CrN is not fully resolved and where literature reports are not entirely consistent. The aim is to clarify whether these parameters favor γN formation or CrN precipitation, and how the resulting microstructure affects localized corrosion behavior in chloride media. To achieve this, the study evaluates microstructural changes, surface hardness, and localized corrosion parameters, including cyclic polarization and critical pitting temperature (CPT), in both untreated and nitrided conditions.

## 2. Materials and Methods

The material studied was Inconel 718 (UNS N07718) alloy, supplied as a cylindrical bar of 146 mm in diameter, already solubilized at 1089 °C for one hour, followed by aging at 788 °C for seven hours. No further heat treatment was performed. The alloy was sectioned into 5 mm thick disks using wire electro-discharge machining (Eurostec Comércio de Máquinas e Acessórios Ltda., Caxias do Sul, RS, Brasil) and further cut into 22 × 11 mm specimens by waterjet cutting Prime Pro 3D 4020 (Technos Prime, Santo André, São Paulo, Brazil) and precision grinding (Buehler Ltd., Lake Bluff, IL, USA). The nominal chemical composition of the alloy is presented in Table 1, which was determined in previously published research [4].

### 2.1. Continuous Low-Temperature Plasma Nitriding (CN)

Before any plasma treatment, all samples were ground sequentially using SiC papers (up to 1500 grit), followed by mechanical polishing with a 1 μm diamond suspension until a mirror finish was achieved. Prior to being introduced into the plasma reactor, all faces of the samples were cleaned with acetone and subsequently immersed in an ultrasonic bath in a 10% ethanol solution. They were then dried using forced convection air. Afterward, the samples were immediately introduced into a cylindrical pulsed cold-wall DC reactor, of the same type used in previous investigations (Ø300 mm × 300 mm) [4,20].

The treatment conditions were based on previous research on nitriding of austenitic matrix alloys [20,21]. A preliminary sputter-cleaning step was performed using a 50% H_2_ and 50% Ar gas mixture (200 sccm) at 300 ± 5 °C for 20 min at 500 V (peak voltage) and 3 Torr, to remove surface oxides and promote surface activation. After sputter-cleaning, continuous low-temperature plasma nitriding was conducted at 425 ± 2 °C for 2 h under 3.75 Torr and a peak voltage of 500 V. The gas mixture consisted of N_2_-H_2_-Ar with flow rates of 50 sccm, 50 sccm and 100 sccm, respectively. Nitrogen served as the active species for nitriding, hydrogen contributed to surface reduction and cleaning, and argon helped stabilize the discharge and maintain consistent ion bombardment. Temperature was monitored using a K-type thermocouple in direct contact with the cathodic table. The temperature was controlled by adjusting the time the Pulse Width Modulation-controlled source was on (t_on_) and off (t_off_). The t_off_ was held constant at 200 µs, while t_on_ was varied 50 ± 10 µs. Upon completion of the treatment, specimens were cooled in a hydrogen flux (200 sccm, 7 Torr) to temperatures below 100 °C before being removed from the chamber. The samples were removed from the reactor, cleaned with acetone, and stored in a vacuum desiccator until they were characterized.

Several previous studies determined that plasma nitriding increases the roughness of Inconel 718 samples, and this could affect wear and corrosion performance [12,13,15,16,17]. Therefore, in this work, non-nitrided samples (named untreated: UN) were subjected to the sputtering process to obtain topographic characteristics comparable to those of nitrided surfaces (named continuous nitrided: CN) but without thermochemical treatment.

### 2.2. Electrochemical Tests

For the electrochemical tests, the samples were embedded in epoxy resin, exposing only the 2.42 cm^2^ nitrided (CN) or sputter-cleaned (UN) surface to the electrolyte. Before the electrochemical tests, the samples were ultrasonically cleaned for 5 min in a 10% *v*/*v* ethyl alcohol solution. They were then dried with warm-air forced convection.

All electrochemical tests were performed using a Gamry Reference 620 potentiostat and (Gamry Instruments, Inc., Warminster, PA, USA) a conventional three-electrode cell with a platinum counter electrode and a saturated calomel electrode (SCE) (Gamry Instruments, Inc., Warminster, PA, USA) as reference. All the potentials quoted in this work refer to the SCE. At least three independent measurements were conducted for each surface condition (UN and CN), and the results are presented as the average and standard deviation.

#### 2.2.1. Cyclic Polarization Test

Table 2 summarizes the test conditions. The corrosion parameters in the Tafel region (corrosion potential (E_corr_), corrosion rate, and corrosion current density (i_corr_)) were determined using the ECHEM ANALYST software (Version 7.10) in accordance with the ASTM G102 standard [22]. Other corrosion parameters, such as passivation current density (i_pass_), pitting potential (E_pit_), and repassivation potential (E_R_) were determined according to the literature [23].

#### 2.2.2. Critical Pitting Temperature (CPT) Test

CPT measurements were performed in accordance with ASTM G150-18 [27] for three independent trials for each condition. The initial temperature of 4 °C was maintained for 10 min. Then, a potential of +700 mV vs. SCE was applied, and after one minute, heating was started at a rate of 1 °C/min. Gas purging (with nitrogen) and potentiostatic polarization were maintained throughout the duration of the test. A 400 mL solution volume was used (1 mol/L NaCl), contained in a flushed port cell of a circular double-walled glass chamber to facilitate heating by an external recirculating heating bath. Because the embedded sample was immersed in the solution and a thermocouple was positioned as close as possible to the sample surface, this temperature was considered and registered rather than that of the heating bath. The CPT was defined as the lowest temperature at which the current density exceeded 100 μA/cm^2^ and remained above this threshold for at least 60 s, as specify by the ASTM G150 standard [27].

### 2.3. Surface Characterization Techniques

X-ray diffraction (XRD) (Shimadzu, model XRD-7000, Shimadzu Corporation, Tokyo, Japan) was performed using Cu-Kα radiation (λ = 1.54060 Å, 30 kV, 30 mA) in grazing-incidence mode at a fixed incident angle of 3°, over a 2θ range of 30–80°. Phase identification was performed using the PDF-4+ database, enabling detection of γ matrix reflections and CrN peaks associated with nitrogen uptake. The identification of the γ_N_ peaks was based on previous literature on this phase in the Inconel 718 alloy [11,15,16,17,19,28].

Microhardness measurements were performed using a Shimadzu HMV-2 Vickers tester (Shimadzu Corporation, Kyoto, Japan) (50 gf load, 10 s dwell) following ASTM E92-23 [29]. Hardness measurements were performed on the top surface of the samples, specifically on the nitrided surface (CN) and the cathodic sputtering surface (UN). A matrix of parallel lines spanning the entire width of the samples (11 mm) was used, with indentations spaced 1 mm apart. For the UN condition, 40 indentations were made, arranged in 4 lines of 10 indentations each. For the CN condition, 60 indentations were made, arranged in 6 lines of 10 indentations each. This was selected to evaluate the hardness distribution across the specimen width, which helps identify potential plasma edge effects characteristic of plasma treatments.

The roughness of the samples was measured on mirror-finished samples, after sputtering (UN samples) and nitriding (CN samples) using a Mahr Marsurf PS10 contact profilometer (MAHR GmbH, Göttingen, Germany), equipped with a 2 μm standard probe. The profiles were acquired with a cut-off of 0.25 mm and an evaluation length of 1.25 mm. A total of 15 measurements were performed for each condition on different samples to ensure the representativeness of the results.

Optical microscopy was carried out using an Olympus BX51M (Olympus BX51RF, Olympus Corporation Shinjuku Monolith, Tokyo, Japan) to assess the nitrided layer thickness and examine the corrosion pit morphology. Cross-sections were electrolytically etched with 10% oxalic acid solution (5 V, 10–15 s) to reveal the microstructure.

Scanning electron microscopy (SEM) was used to improve the characterization of the corroded surface. Imaging was conducted in a TESCAN LYRA3 (Tescan Lyra 3, TESCAN ORSAY HOLDING, Brno—Kohoutovice, Czech Republic) at an accelerating voltage of 20 kV using both secondary-electron (SE) and backscattered-electron (BSE) detectors. EDS point analyses and elemental mapping were performed to evaluate elementary chemical compositions in regions associated with corrosion damage.

## 3. Results

### 3.1. X-Ray Diffraction and Thickness Layer Analysis

**Figure 1:**
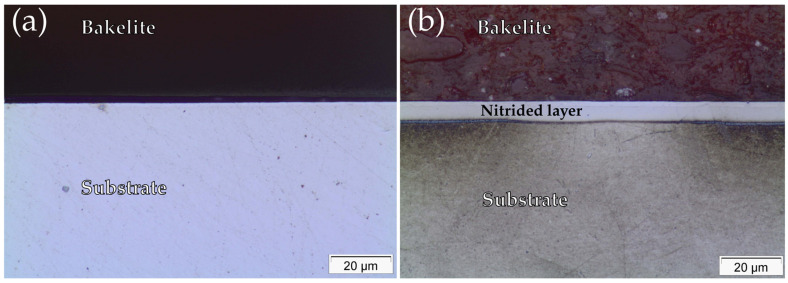
Optical micrograph of the untreated sample (**a**) and continuous nitrided sample (**b**) at 100× magnification.

Cross-section OM micrographs of untreated (UN) and continuous plasma nitrided (CN) samples are shown in Figure 1. As shown in Figure 1a, the untreated Inconel 718 specimen, which was subjected to the sputtering process, did not form a detectable surface layer, consistent with the absence of thermochemical treatment. In contrast, the CN sample (Figure 1b) exhibited a well-defined, continuous layer of a lighter color than the substrate, standing out from the substrate; the approximate thickness values of this layer ranging from 6.1 to 6.7 µm.

**Figure 2:**
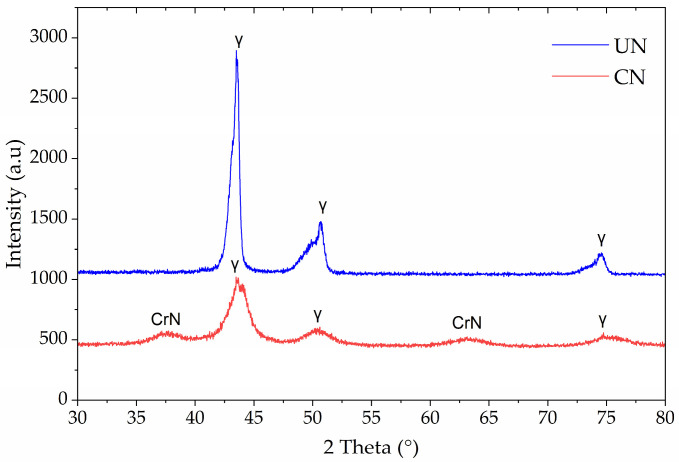
X-ray diffraction patterns of untreated (UN) and continuous nitrided (CN) Inconel 718 samples.

Figure 2 shows the X-ray diffraction patterns of untreated (UN) and continuous nitrided (CN) Inconel 718. For the untreated sample, sharp peaks corresponding to the γ-phase of the FCC structure dominated the pattern, notably at ~43.5° and 50.7° (2θ), consistent with the base alloy microstructure. No secondary nitride phases were detected, confirming the stability of the γ-matrix under the sputtering pre-treatment.

In contrast, the CN sample displayed CrN diffraction peaks at ~38° and ~64° (2θ), in addition to the γ-phase reflections. The absence of N-expanded austenite (γ_N_) peaks and the appearance of CrN confirms that nitrogen incorporation at 425 °C for 2 h favored chromium nitride precipitation rather than expanded austenite stabilization. Also, it is observed that the austenite peaks exhibit increased full width at half maximum (FWHM), as was also observed in other investigations of plasma-nitrided Inconel 718, [4,19].

### 3.2. Microhardness Test Results

**Figure 3:**
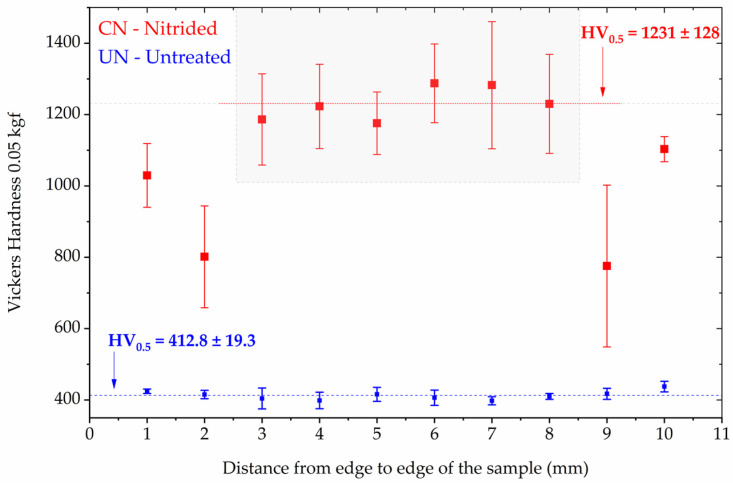
Vickers microhardness distribution (0.05 kgf) as a function of distance from the sample edges for untreated (UN) and continuous nitrided (CN) Inconel 718 specimens. The error bars represents the standar deviation at each location. The gray rectangle indicates the region where the hardness values were more homogeneous for the CN condition.

Microhardness testing was performed on the untreated (UN) and continuous nitrided (CN) Inconel 718 samples using a 0.05 kgf load. Figure 3 presents the results of the indentation matrix performed on the surface of the samples. The results are presented as the average and its standard deviation (error bars) for each position of the hardness profile measured across the width of the samples (11 mm).

The UN condition displayed a relatively uniform profile (Figure 3), with a hardness value of HV_0.5_ = 412.8 ± 19.3. In contrast, the CN condition exhibited a substantial increase in hardness, with the highest hardness values in the central region (gray area of Figure 3) being HV_0.5_ = 1231 ± 128. However, pronounced non-uniformity was observed across the sample width, with near-edge values dropping to as low as ~800 HV in mean. The lateral drop is consistent with edge-effect field distortions during plasma treatment, which leads to uneven ion flux and nitrogen uptake near borders [30].

It is necessary to highlight for the reader that the CN’s hardness values do not represent the intrinsic hardness of the nitrided layer. Due to the indentation conditions applied, the measurements are influenced by the Inconel 718 substrate; therefore, they are substrate dependent. Moreover, the nearly threefold increase in hardness compared to the untreated state is consistent with previous reports of CrN strengthening in Inconel 718 treated at similar temperatures [4,12,31].

### 3.3. Surface Roughness

Table 3 presents the most representative height roughness parameters for the topography of each surface condition: Ra (Arithmetic Mean Roughness), Rq (Root mean square deviation), and Rz (Max Height of profile). The values for the mirror surface finish are shown as a comparison to the topography of the samples before they were introduced into the reactor.

First, the significant effect of the sputtering process alone on surface topography is observed. This is evident when comparing the roughness between polished surfaces (mirror finished) and non-nitrided surfaces (UN samples) subjected to the sputtering process. Then, considering the dispersion of results, it is observed that the plasma nitriding process itself did not generate such significant topographic changes from the surfaces in the sputter condition. These results are relevant, as other investigations have only compared the topographic changes before and after plasma nitriding treatments of Inconel 718, finding statistically significant increases in roughness height parameters in most cases [4,12,13,15,16]. Importantly, the modest changes in roughness are insufficient to explain the substantial deterioration in localized corrosion resistance observed later in this study, indicating that surface roughness alone is unlikely to account for the drastic deterioration in localized corrosion resistance between UN and CN samples.

### 3.4. Cyclic Polarization Results

**Figure 4:**
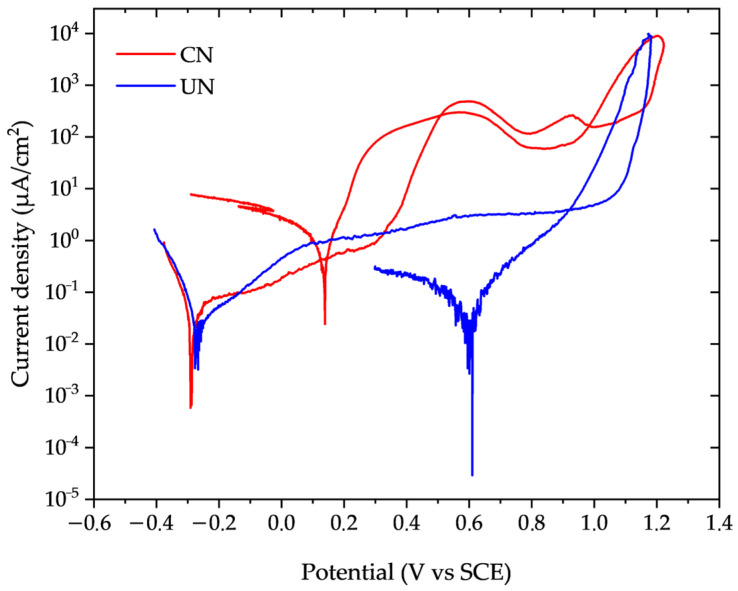
Potentiodynamic polarization curves of untreated (UN, blue) and continuous nitrided (CN, red) Inconel 718 samples in 3.56% NaCl solution at 22 ± 2 °C (Scan rate: 1 mV/s).

Representative cyclic potentiodynamic polarization curves for the untreated (UN) and continuous nitrided (CN) conditions are presented in Figure 4. The relevant corrosion parameters extracted from these curves are summarized in Table 4.

The corrosion current density (i_corr_) of the CN condition nearly doubled that of the untreated sample, and the calculated corrosion rate increased accordingly. Nitrided samples showed a deterioration of passivity at potentials between 0.30 and 0.40 V, as indicated by the increase in passivation current density. This increase in current density was two orders of magnitude higher for CN, compared to UN, which kept the passivation current density practically constant, between 1 and 10 µm/cm^2^. Considering the standard deviation of the measured values (Table 4), both the pitting and repassivation potentials were slightly more anodic for the nitrided sample compared to the untreated one. However, these increases were below 10%, indicating that nitriding essentially does not significantly alter the onset of localized corrosion under the evaluated conditions (3.56% NaCl, at 22 ± 2 °C).

### 3.5. Surface Characterization After Cyclic Polarization Tests

**Figure 5:**
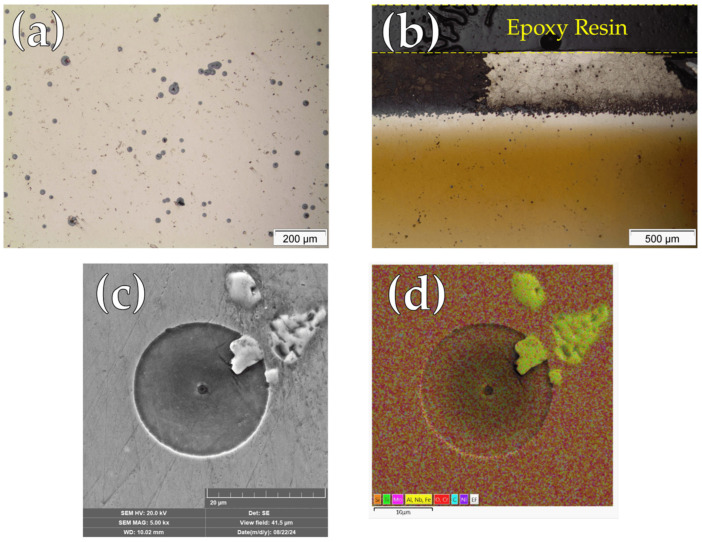
Micrographs of untreated Inconel 718 after cyclic polarization test: (**a**) Optical micrograph at the central region; (**b**) Optical micrograph at the edge region; (**c**) high-magnification SEM micrograph of a pit; (**d**) SEM/EDS elemental mapping.

Optical and SEM/EDS microscopy techniques were employed to visually assess the corrosion morphology of Inconel 718 specimens after cyclic polarization testing, examining both central and edge regions of the surface samples. In the central region of untreated samples (Figure 5a), there is clear evidence of localized corrosion in the form of pitting. The pits appeared generally circular and randomly distributed across the surface, indicating susceptibility to chloride-induced attacks. The edge region (Figure 5b) revealed additional surface alterations, which may be attributed to localized electric field intensification during sputtering. At higher magnification (Figure 5c), the pits showed a central crater surrounded by a well-defined circular dissolution zone. Figure 5d is a SEM/EDS mapping of Figure 5c, in which it is evident that the pits nucleated randomly and not preferentially around the Nb-rich precipitates, as reported by Wei and Zheng 2020 [32].

**Figure 6:**
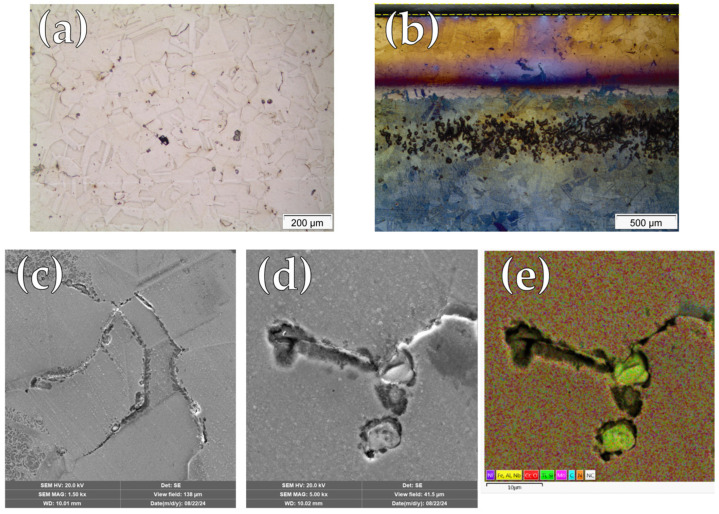
Micrographs of continuous nitrided (CN) sample after cyclic polarization test: (**a**) Optical micrograph at the central region; (**b**) Optical micrograph at the edge region; (**c**) high-magnification SEM micrograph; (**d**) Detail of localized attack; (**e**) SEM/EDS elemental mapping.

Figure 6 presents micrographs of the nitrided surfaces after cyclic polarization testing. In the central region (Figure 6a), localized corrosion sites with different geometries and randomly distributed on the surface can be observed. On the other hand, the edge region (Figure 6b) displayed a dense accumulation of pits, highlighting the pronounced vulnerability of areas subjected to plasma edge effects. This concentration is likely associated with local variations in plasma exposure and electric field distribution during treatment, which may have promoted passive film instability [30].

Figure 6c presents a SEM micrograph revealing localized corrosion along the grain boundaries of the microstructure. Higher-magnification observations of these regions (Figure 6d) indicate that, in addition to intergranular corrosion, localized attack preferentially develops in the vicinity of precipitates enriched in Fe, Al, Nb, and Ti, as identified by SEM/EDS elemental mapping (Figure 6e).

### 3.6. Critical Pitting Temperature (CPT) Results

**Figure 7:**
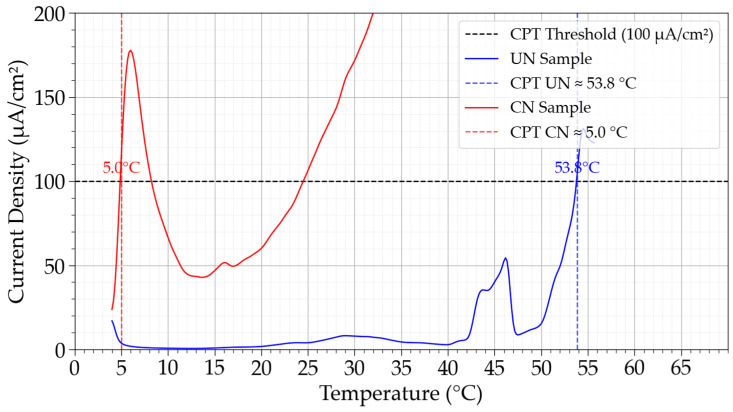
Critical Pitting Temperature (CPT) determination for untreated (UN) and continuous nitrided (CN) Inconel 718 samples. Vertical lines indicate the CPT values for each condition according to ASTM G150 criteria.

Figure 7 presents the current density curves as a function of temperature for the determination of the Critical Pitting Temperature (CPT). The untreated samples exhibited stable passive behavior up to elevated temperatures, with CPT values of 58.3, 50.5 °C, and 34.5 °C in three independent trials, yielding an average of 46.3 ± 10.3 °C. In contrast, the CN condition showed markedly lower resistance, with CPT values of 5.0 °C, 16.5 °C, and 5.2 °C, averaging only 8.8 ± 6.6 °C. This represents a reduction of nearly 37.5 °C compared to the untreated state. This high difference underscores the detrimental impact of continuous nitriding performed in this work on temperature-dependent localized corrosion resistance.

## 4. Discussion

### 4.1. Microstructural and Surface Modifications

The optical micrographs in Figure 1 reveal that a nitrided surface layer was formed in the CN samples, with thickness values ranging from 6.1 to 6.7 µm. The absence of γN (expanded austenite) in the XRD patterns (Figure 2) confirmed that, under the applied nitriding conditions, chromium nitride precipitation was the dominant nitrogen incorporation mechanism.

This microstructural change was accompanied by a nearly threefold increase in hardness compared with the untreated alloy, from ~413 HV to values exceeding 1200 HV. Similar improvements in hardness linked to CrN precipitation have been reported in prior studies on Inconel 718 and related alloys [4]. However, hardness profiles revealed significant variability, with edge regions showing reductions of more than 30% compared to the average value of the central region. This indicates that continuous plasma exposure intensifies the “edge effect,” producing non-uniform ion flux and nitrogen distribution, as previously described in the plasma nitriding of Ni- and Fe-based alloys [4,30].

According to a recent review on Inconel 718 [14] temperatures below 450 °C typically promote the formation of layers primarily composed of γN and CrN during gaseous, plasma, and liquid nitriding. In contrast, treatments at temperatures ≥ 450 °C typically do not produce the expanded phase, because γN tends to decompose into CrN + γ [12,13,15,19]. Based on this criterion, the absence of γN in Figure 2 appears to contrast with the trends generally reported in the literature. Only two studies, Jing et al. and Tao et al., have specifically evaluated nitriding at 425 °C [13,19]. Both authors reported CrN precipitation only after prolonged treatments (≥16 h). The increase in treatment time at 425 °C may allow partial relaxation of the γ_N_ lattice and enable short-range Cr diffusion, so that the system lowers its free energy not by stabilizing a highly strained γ_N_, but by initiating the decomposition of γN toward a γ + CrN mixture accompanied by local chromium depletion [13,19].

However, the results were different for treatment times ≤ 8 h at 425 °C [13,19]. For example, Jing et al. reported that 4 and 8 h of liquid nitriding treatments produced nitrided layers whose microstructure remained similar to the untreated alloy, consisting mainly of the γ-FCC matrix and showing no evidence of CrN or S-phase formation. They attributed the broader full width at half maximum (FWHM) of the γ-FCC peaks, also observed in Figure 2 for the CN condition, to an initial complex stress state caused by nitrogen incorporation into the γ lattice, which was insufficient to form the expanded phase fully [19]. In contrast, Tao et al. reported the formation of a γ + γN layer after 4 h of plasma nitriding at 425 °C, differing from the findings of the present work [13].

The predominance of CrN over γ_N_ in the nitrided layer in the present study can be tentatively rationalized by considering the thermodynamic and kinetic competition between these phases in Ni–Cr alloys, promoted by ion flux dependent on plasma nitriding parameters (detailed in Section 2.1). According to Kovači et al. and Aw, Batchelor, and Loh [7,15], this may occur when the sputtering–redeposition process is inefficient, enabling a rapid reaction between nitrogen and chromium that promotes CrN formation over γ_N_. This mechanism is consistent with the well-known challenges of nitriding Ni-based alloys, where nitrogen exhibits substantially higher solubility in chromium than in nickel, as extensively documented by Williamson, Davis, and Wilbur [33]. Studies on Inconel 718 and other Cr-containing alloys indicate that, at temperatures around 400 °C, nitrogen diffuses much faster than substitutional elements such as chromium, leading initially to the formation of a metastable, nitrogen-supersaturated expanded austenite (γ_N_) that can provide simultaneous improvements in hardness and corrosion resistance [11,19]. As temperature or treatment time are increased, the low equilibrium solubility of nitrogen in the FCC matrix, together with the high nitride-forming tendency of chromium, promotes CrN nucleation [11]. With this basis, it is reasonable to hypothesize that, at 425 °C, both the thermodynamic driving force for chromium nitride formation becomes more significant than at 400 °C.

In light of these observations, the precipitation of CrN at 425 °C in the present work may therefore be understood as a plausible consequence of this thermodynamic/kinetic competition imposed by the plasma nitriding conditions. The γ peaks identified in CN (Figure 2) were broader, asymmetric, and less intense than those of untreated IN718, indicating incipient nitrogen incorporation into the FCC structure. However, this diffusion was clearly insufficient to promote complete γ_N_ formation, instead of favoring CrN precipitation. Consequently, the nitriding parameters selected in this study appear suboptimal for Inconel 718, despite having previously produced expanded phases without CrN in an austenitic stainless steel (ISO 5832-1) [20].

### 4.2. Electrochemical Behavior

The corrosion parameters obtained from cyclic polarization curves revealed only minor differences between the evaluated conditions. Notably, nitriding led to an approximately twofold increase in the corrosion rate compared to the untreated condition. In contrast, the pitting (E_pit_) and repassivation (E_R_) potentials were slightly more anodic for the nitrided samples (CN). However, despite these marginally higher potentials, the CN condition exhibited passive current densities nearly two orders of magnitude higher than those of the untreated material (UN). In contrast, the UN condition maintained a stable and low passive plateau.

The results obtained from cyclic polarization (CP) and critical pitting temperature (CPT) tests are consistent with each other. At an applied potential of +0.7 V, employed during CPT measurements, nitrided samples exhibited current densities approximately two orders of magnitude higher than the passive current density of untreated specimens, as inferred from the cyclic polarization curves. This agreement reinforces the interpretation that nitriding did not improve, but somewhat impaired, the corrosion resistance under the investigated conditions.

A clear change in the localized corrosion mechanism was also observed, shifting from pitting corrosion in the untreated condition (Figure 5) to predominantly intergranular attack and preferential localized corrosion around precipitates in the nitrided samples (Figure 6). Notably, the same corrosion features were identified on specimens after CPT testing, confirming the reproducibility of the observed degradation mechanisms in chlorine-containing electrolytes.

Chromium depletion in several regions of the matrix, resulting from CrN precipitation, is likely a key factor contributing to the deterioration of corrosion resistance. Similar behavior has been reported in the literature for nitrided layers composed predominantly of CrN, without γ_N_ formation [13,28,34]. Additionally, preferential pit nucleation at grain boundaries of nitrided IN718 surfaces has been previously reported by Xue et al. [17] for liquid nitriding treatments, as seen in Figure 6c. According to these authors, the accumulation of lattice defects, such as dislocations at grain boundaries, locally lowers the corrosion potential relative to adjacent grains, promoting the formation of galvanic microcells [17].

Furthermore, nitrided samples exhibited localized attack along the periphery of precipitates enriched in Fe, Al, Nb, and Ti, as identified by SEM/EDS elemental mapping (Figure 6d,e). These precipitates were already present in the untreated material (Figure 5c,d), and given their compositional disparity with the matrix, galvanic effects at the matrix–precipitate interfaces could be expected, as confirmed by Wei and Zheng [32]. However, such galvanic interactions did not initiate pit nucleation in the untreated condition, as evidenced in Figure 5c. Therefore, it can be inferred that nitriding increased the electrochemical potential differences between the matrix and these precipitates, leading to the formation of galvanic microcells at these interfaces. This behavior is likely exacerbated by the reduced corrosion resistance of the matrix due to chromium depletion associated with CrN precipitation, as discussed previously.

The CPT behavior confirms that the passive film on the UN condition is thermally stable and capable of resisting the synergistic effects of temperature and chloride ions. In sharp contrast, the nitrided condition exhibited a loss of passivity, with an average CPT near ambient temperature (8.8 ± 6.6 °C). The difference of ~37.5 °C quantifies the penalty caused by the nitriding treatment, which, regardless of the application and service conditions of Inconel 718, probably does not compensate for the gain in hardness obtained.

The CPT values obtained here place the untreated Inconel 718 at an intermediate level of localized corrosion resistance when compared with other Ni-rich alloys evaluated by ASTM G150-type methodologies. For example, electrochemical CPT tests in 1 mol/L NaCl on CoCrFeNiTa_x_ high-entropy alloys following ASTM G150 [27] have reported critical pitting temperatures well above room temperature, reflecting their passive behavior under aggressive chloride loading [35]. Similarly, Inconel 625 claddings tested in NaCl solutions showed CPT values exceeding those of stainless steels, confirming the intrinsically high pitting resistance of Ni–Cr–Mo alloys when chromium and molybdenum remain in solid solution [36]. Frazão et al. further emphasized that many nickel alloys exhibit CPT values approaching or exceeding the boiling point of 1 mol/L NaCl, which can even limit the direct applicability of the standard ASTM G150 [27] procedure for the highest-alloyed Ni–Cr–Mo systems [37]. With the previous information, the reduction in CPT from 46.3 °C (UN) to 8.8 °C (CN) observed in the present study clearly positions plasma-nitrided Inconel 718 far below the performance typically associated with corrosion-resistant Ni-based materials.

The severity of this deterioration is consistent with the microstructural changes induced by continuous low-temperature plasma nitriding at 425 °C, namely the formation of a CrN-rich surface layer. Together, these changes degrade the stability of the passive film in chloride electrolytes. Consequently, although the CN condition provides substantial gains in hardness and may offer improved wear performance, as shown in other studies [15,38], the pronounced drop in CPT implies a significantly reduced safety margin against pitting corrosion for components operating in marine or chloride-containing environments. Any practical implementation of continuous plasma nitriding for Inconel 718 under the process conditions employed in this study (Section 2.2) must therefore carefully balance the achieved mechanical enhancements against the degradation in localized corrosion resistance revealed by the CPT measurements.

### 4.3. Trade-Offs and Implications

Taken together, the results highlight a critical trade-off: continuous plasma nitriding markedly enhances hardness but at the expense of corrosion resistance in Cl-containing environments. The dual role of nitrogen incorporation is evident as beneficial when forming expanded austenite, but detrimental when favoring CrN precipitation. These findings stress the importance of carefully controlling plasma process parameters to mitigate the detrimental effects of CrN formation. They also emphasize the importance of employing different electrochemical analyses, as relying on a single test type could mask subtle yet critical degradations in passive film stability. The results presented here are limited to the evaluated conditions of concentration and temperature of the NaCl solutions used; however, from an engineering perspective, these results indicate that the nitriding conditions used may be viable for applications where case hardness is paramount and chloride exposure is minimal.

## 5. Conclusions

This study evaluated the effect of continuous low-temperature plasma nitriding (425 °C, 2 h) on Inconel 718, with emphasis on the balance between hardness improvement and corrosion resistance in chloride-containing environments. The results demonstrate that the selection of plasma nitriding temperature and treatment time alone does not entirely govern the solid solution strengthening mechanism typically sought in this type of surface treatment. Accordingly, the findings contrast with the existing literature and suggest that the imposed thermochemical conditions did not promote sufficiently efficient nitrogen diffusion to enable the formation of nitrogen-expanded austenite. Instead, a surface layer predominantly composed of CrN was formed, providing a hardness approximately three times higher than that of the untreated material. Consequently, the electrochemical behavior was dominated by the effects associated with chromium nitride formation, resulting in passivity degradation, a twofold increase in the uniform corrosion rate, and a reduction of more than 30 °C in the critical pitting temperature for the nitrided surfaces.

## Figures and Tables

**Table 1:** Percentages (wt.%) of the IN 718 alloy main elements. Taken from [4].

Element	Ni	Cr	Fe	Nb	Mo	Ti	Al	Co
wt.%	53.45	18.28	18.81	4.94	2.83	0.93	0.50	0.23

**Table 2:** Cyclic Polarization Test Parameters.

Parameter	Conditions	Reference
Open Circuit potential (OCP)	Recorded for one hour	ASTM G61-86 [24]
Scan Rate	1 mV/s	[25,26]
Potential Range	Start at -0.20 V relative to OCP; reverse at 5 mA/cm^2^	ASTM G61-86 [24]
Electrolyte Solution	Volume of 200 mL of 3.56% m/m NaCl solution (pH = 6.25–7.00) deoxygenated for 30 min with nitrogen gas before testing, maintained at 22 ± 2 °C.	ASTM G61-86 [24]

**Table 3:** Surface roughness parameters (Ra, Rq, Rz) for untreated (UN) and continuously nitrided (CN) Inconel 718 samples. Values represent mean ± standard deviation (μm). Initial surface roughness is presented for comparison purposes.

Condition	Ra (μm)	Rq (μm)	Rz (μm)
**Mirror surface finish (initial surface)**	0.013 ± 0.002	0.025 ± 0.002	0.176 ± 0.004
**UN**	0.05 ± 0.01	0.06 ± 0.02	0.26 ± 0.06
**CN**	0.06 ± 0.01	0.08 ± 0.02	0.31 ± 0.06

**Table 4:** Comparative Corrosion Parameters of Inconel 718 Before and After Plasma Nitriding.

Parameter	Untreated (UN)	Continuous Nitrided (CN)
*E_corr_* (V)	–0.258 ± 0.022	–0.267 ± 0.026
*i_corr_* (μA/cm^2^)	0.0303 ± 0.0005	0.0618 ± 0.0013
*Corrosion rate* (mm/year) × 10^−4^	3.06 ± 0.09	6.37 ± 0.01
*E_pit_* (V vs. SCE)	0.94 ± 0.02	0.99 ± 0.01
E_R_ (V vs. SCE)	1.07 ± 0.01	1.15 ± 0.03

## Data Availability

The original contributions presented in this study are included in the article. Further inquiries can be directed to the corresponding author.
